# Analyzing the Effects of Intrauterine Hypoxia on Gene Expression in Oocytes of Rat Offspring by Single Cell Transcriptome Sequencing

**DOI:** 10.3389/fgene.2019.01102

**Published:** 2019-11-12

**Authors:** Ting Li, Yang Liu, Shaojie Yue, Zhengchang Liao, Ziqiang Luo, Mingjie Wang, Chuanding Cao, Ying Ding, Ziling Lin

**Affiliations:** ^1^Deparment of Pediatrics, Xiangya Hospital, Central South University, Changsha, China; ^2^Department of Physiology, Xiangya School of Medicine Central South University, Changsha, China

**Keywords:** intrauterine hypoxia, oocytes, single cell sequencing, transcriptome sequencing analysis, multigenerational inheritance

## Abstract

Intrauterine hypoxia is one of the most frequently occurring complications during pregnancy, and the effects of antenatal hypoxia in offspring are not restricted to the perinatal period. Previous studies have reported on this phenomenon, which is usually described as multigenerational or transgenerational inheritance. However, the exact mechanism of this type of inheritance is still not clear. Therefore, in the present study, we investigated the alteration in the gene expression of oocytes, derived from intrauterine hypoxia rats and their offspring, by transcriptome sequencing. Our results showed that 11 differentially expressed genes were inherited from the F1 to F2 generation. Interestingly, these differentially expressed genes were enriched in processes predominantly involved in lipid and insulin metabolism. Overall, our data indicated that alteration in the gene expression of oocytes may be associated with some metabolic diseases and could potentially be the basis of transgenerational or multigenerational inheritance, induced by an adverse perinatal environment.

## Introduction

Intrauterine hypoxia, one of the most frequently occurring complications during pregnancy, is often caused by eclampsia or gestational diabetes. Maternal exposure to a hypoxic environment due to a high altitude usually results in vital damage to the heart (Clemmer and Telford, 1966) and lung (Gortner et al., 2005) of the fetus. In addition, since the last century, there has been a growing concern about the long-term effects of intrauterine hypoxia toward offspring (Silverman et al., 1998). The hypothesis “Fetal origins of adult disease,” proposed by Dr. Barker in 1993 (Barker et al., 2002), identified that maternal malnutrition could lead to an increased risk of cardiovascular diseases and changes in the metabolic status of the offspring. More recently, additional evidence has indicated that adverse intrauterine environments caused by air pollution (Bolton et al., 2014), maternal stress (Weinstock, 2005), or intrauterine hypoxia (Li et al., 2017) can lead to long-term harmful changes in the offspring, and these changes can be passed to the future generation (Zambrano et al., 2005) despite the offspring not being exposed to the pivotal perinatal environment. Broadly, transgenerational inheritance is defined as transmission of genetic information through different generations (Babenko et al., 2015). Interestingly, based on classical genetics, the genetic information is determined by the DNA sequence, and all organisms have precise genome repair and protection mechanisms to protect against the effects of genome instability. This implies that the majority of environmental factors are not able to cause changes directly in the DNA sequences and that the chances of any transgenerational or multigenerational inheritance due to epigenetic modification are minimal. Although the underlying mechanism of transgenerational inheritance is still unclear, recent studies have proposed that phenotypic changes may work through epigenetic control (Gluckman et al., 2007). In 2008, a study by Crews et al. concluded that heritable modifications are those that occur in the germline (Crews, 2008). Notably, germ cells are involved in the transmission of genetic information between generations, and information regarding any epigenetic modifications in them due to the environment can lead to the inheritance of the acquired phenotypes. The zygote, the female germ cell post fertilization with a sperm cell, forms the basis of life as a fetus. The fetal period is not only critical for individual formation and organ development, but it is also an important period for the differentiation of primordial germ cells. In the meantime, epigenetic modifications also result in large-scale erasure and reconstruction during this period. Thus, any exposure to adverse environmental stress during fetal gametogenesis can have long-term effects in the offspring. Therefore, transcriptomic analysis can be a key part of understanding the mechanism of intergenerational inheritance. Previous transcriptomic studies using different organs for analyzing intergenerational or transgenerational inheritance were unable to explain the inheritance phenomenon completely (Zhang et al., 2015; Nguyen et al., 2017). Interestingly, recent advances in single-cell RNA sequencing (RNA-seq) technology have provided an opportunity to study the gene expression pattern in oocytes. This is the first study using RNA-seq to analyze alteration in gene expression among oocytes of offspring derived from intrauterine hypoxia-treated Sprague Dawley rats. In this study, our analyses of the changes in the gene expression among F1 and F2 generations of oocytes demonstrated that some alteration could be inherited; thus, it could be the fundamental basis of the intergenerational or transgenerational inheritance phenomenon.

## Materials and Methods

### Animals

Female (200–250 g) and male Sprague-Dawley rats (300–350 g) were purchased from the Animal Center of Central South University, Changsha, China. The rats were housed in a controlled environment with temperature of 22°)C and a 12:12 h light/dark cycle, and they were allowed *ad libitum* access to food and water. The day with visible vaginal plugs was considered as day 0 of gestation (G0). Later, the pregnant rats at G19 were randomly divided into two groups (n = 6); (i) air-control group, in which the rats were maintained under normoxic conditions (21% O2)), and (ii) hypoxia group, in which the rats were treated under hypoxic conditions in a plexiglass chamber with FiO2) = 9.5%–11.5% for 8 h/d during G19 and G20. The hypoxic environment was maintained by using nitrogen gas, as reported previously by our team, and the oxygen concentration was strictly monitored by an oxygen analyzer every 30 min (Li et al., 2017). The reasons why we chose the “Alternating hypoxia–normoxia” animal model is because prolonged hypoxia could lead to the reduction of the maternal appetite and thus it is not clear whether hypoxia or nutrient limitation was responsible for fetal growth restriction according to previous study (Schwartz et al., 1998). Additonally, our pre-experiment results showed that prolonged hypoxia could significantly increase the mortality rate of the pregnant rat. What’s more, from the obstetric experience, oxygen inhalation to prevent hypoxia for long periods is a common treatment in pregnant women. All animal procedures were reviewed and approved by the Committee of Research Animal Welfare, Central South University, Changsha, China. After delivery, the newborn rats were classified as the air-control F1 and hypoxia F1 rats, respectively. The newborn rats were regularly fed without any intervention after birth. At 8 weeks of age, four female rats from the air-control and hypoxia F1 groups were randomly selected and crossed with other 8-week-old male rats originating from the same mother and father. After successful mating and pregnancy, the F1 pregnant rats had natural delivery without any intervention. The offspring of the air-control and hypoxia F1 groups were defined as the air-control F2 group and the hypoxia F2 group, respectively. The body weights of all these rats were recorded at postnatal day 3 (PND3), PND7, PND14, PND21, PND28, and 8 weeks.

### Oocyte Preparation

**Figure 1:**
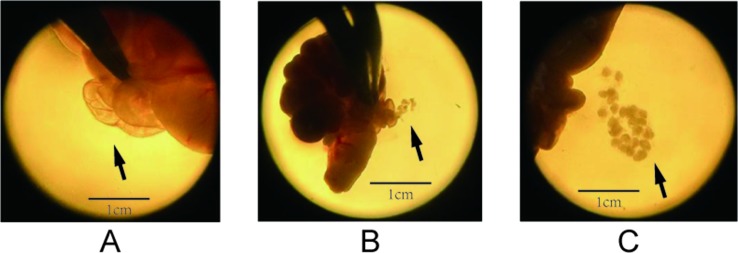
Process of dissecting oocytes under a microscope (1.5× magnification). **(A)** The solid arrows point to the enlarged tubal ampulla, **(B)** the microscopic spur poking ampulla of the fallopian tube, and **(C)** the cumulus–oocyte complex (COC).

**Figure 2:**
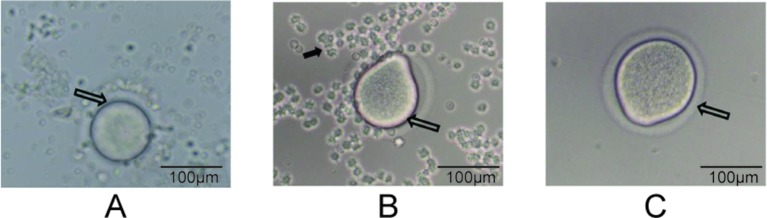
Degranulation process (200× magnification). **(A)** The hollow arrows point to the oocyte of the COC, **(B)** the oocyte during degranulation, and **(C)** a completely prepared oocyte. The solid arrow in panel B points to granulosa cells.

The superovulation technique (Zhang et al., 2007) was used to prepare the oocyte samples for single-cell RNA-seq. At PND28 for the F1 and F2 groups, we randomly selected four female rats from each group. After treatment with pregnant mare serum gonadotropin (PMSG, 20 U) and human chorionic gonadotropin (HCG, 20 U), as described previously (Zhang et al., 2007), the oocytes were ready to be collected. Subsequently, intraperitoneal injection of 10% chloral hydrate (0.3 ml/kg) was performed, and the bilateral uterus, ovary, and oviduct were removed by laparotomy to separate the oocytes under a dissecting microscope. The enlarged ampulla of the fallopian tube was identified under a dissecting microscope, and it was poked to collect cumulus–oocyte complexes. The process of dissecting oocytes was displayed in **Figure 1**. Each cumulus–oocyte complex was transferred to 0.05% hyaluronidase droplets to perform degranulation (the process of degranulation was displayed in **Figure 2**); and after removal of the granules, each oocyte was collected in a polymerase chain reaction tube. We collected three oocytes (one from an individual rat) from every group. In total, there were 24 oocyte samples,which were prepared for the following transcriptome sequencing.

### Single-Cell Transcriptome Sequencing

After preparation of the oocyte from different groups, we used the SMART-Seq *TM*) v4 Ultra *TM*) Low Input RNA Kit for Sequencing (Takara Bio Company), according to the manufacturer’s protocol, which include: SMARTer first-strand cDNA synthesis, full-length ds cDNA amplification by LD PCR, purification and validation of amplified cDNA, and library preparation and sequencing on the Illumina sequencing platform (Illumina HiSeqTM2000). The RNA integrity was evaluated using an Agilent2100 Bioanalyzer (Agilent Technologies, Santa Clara, CA, USA). An RNA integrity number ≥7 was considered as the criterion to select the samples for subsequent analysis. The libraries were prepared using a TruSeq Stranded mRNA LTSample Prep Kit (Illumina, San Diego, CA, USA), according to the manufacture’s instructions. Next, these libraries were sequenced on the Illumina sequencing platform (Illumina HiSeqTM2000), and 125 bp/150 bp paired-end reads were generated.

### Identification of Differentially Expressed Genes

Transcriptome sequencing is a kind of high efficiency methods which could analyze the gene expression from the RNA level. Transcriptional level is the sum of the expression and degradation. The quantification of gene transcript abundance was assessed by values of fragments per kilobase of transcript per million mapped reads calculated using Htseq-count software (Roberts, 2011) and Cufflinks software (Simon et al., 2015). The difference in gene expression among the different groups was calculated using the DESeq (2012) R package (Simon Anders, 2016). A gene was categorized as being differentially expressed if the difference in the expression between groups was >2-fold or <0.5-fold, with a *p* value <0.05. The false discovery rate for every differentially expressed genes (DEGs) is calculated using the Benjaminiand Hochberg method.

### Gene Ontology and Kyoto Encyclopedia of Genes and Genomes Pathway Analysis

The gene ontology (GO) (Hulsegge et al., 2009) method was used to analyze the transcriptomic data. It consists of three parts: biological process, cellular component, and molecular function. In addition, to perform pathway analysis of DEGs, we used the Kyoto Encyclopedia of Genes and Genomes (KEGG) (Minoru et al., 2008) database. The DEGs were uploaded into the Database for Annotation, Visualization, and Integrated Discovery (http://david.abcc.ncifcrf.gov/) and KEGG (http://www.genome.ad.jp/kegg/). The GO and KEGG pathway analyses were performed using the standard enrichment computation method.

### Statistical Analysis

The weight values were expressed as mean ± SD. The statistical analysis was performed with SPSS17.0. Statistical comparisons among the groups were assessed by independent T-test. P-value of <0.05 was considered statistically significant.

## Results

### The Intrauterine Hypoxia Effect on Offspring’s Weight After Birth

In the first week after birth, the body weight of hypoxia rats (F1 generation) was much lower than that in the control group (*p* < 0.01). The weight trend of the F2 generation is similar with the F1 generation. Additionally, the reduction in weight persisted till the 28 days after birth in F2 generation (**Table 1**).

**Table 1:** The intrauterine hypoxia effect on F1 and F2 generation’s weight after birth ( ± *s*, *g*, *n* = 15).

Generation	Group	BW	PND3	PND7	PND14	PND28	PNW8
F1	Air control	7.32 ± 0.70**)	11.32 ± 1.15**)	18.34 ± 1.76	35.60 ± 5.57	94.0 ± 5.03	301.5 ± 18.33
	Hypoxia			16.71 ± 0.39	34.10 ± 1.26	90.70 ± 3.00	298.8 ± 12.11
F2	Air control	7.62 ± 0.09	10.50 ± 0.29	20.55 ± 0.35	37.63 ± 0.68	115.7 ± 2.10	313.5 ± 15.23
	Hypoxia	6.11 ± 0.16**)	8.48 ± 0.31**)	18.01 ± 0.42*)	33.46 ± 0.6*)	95.34 ± 1.79	300.6 ± 10.11

*p < 0.05 vs. air control group.

**p < 0.01 vs. control group.

### Identification of DEGs

**Figure 3:**
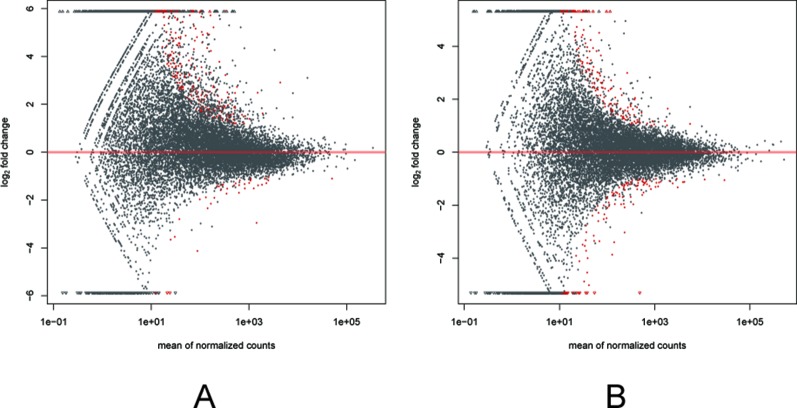
MA plots **(A** and **B)** visualizing the differentially expressed genes (DEGs). The X-axis: Average expression of all samples for comparison after standardization; The Y-axis: log2FoldChange. The red dots represented the DEGs. The red dots above the red line: Up-regulated, below the red line: Down regulated. **(A)** the F1 generation DEGs: air-control versus hypoxia group. **(B)** The F2 generation DEGs: air-control versus hypoxia group.

**Figure 4:**
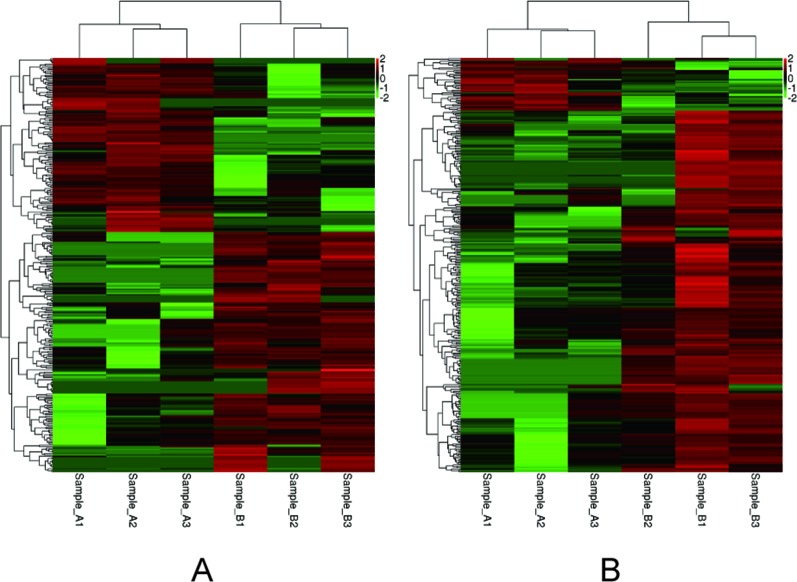
Heat-maps **(A** and **B)** of hierarchical clustering analysis depicting the DEGs. The horizontal ordinate represents the clustering of samples from the air-control and hypoxia groups (F1, F2). The longitudinal coordinates represent the DEGs and the clustering of genes. The red color indicates upregulated genes, while the green color indicates downregulated genes. **(A)** the F1 generation: air-control versus hypoxia group. **(B)** The F2 generation: air-control versus hypoxia group.

Among the 307 DEGs identified between oocytes from the air-control F1 and hypoxia F1 rats, 178 were upregulated while 129 were downregulated (All F1 generation DEGs are listed in **Table S1**). Similarly, 316 DEGs were identified between oocytes from the air-control F2 and hypoxia F2 rats, of which 276 were upregulated and 40 were downregulated (**Figure 3**, all F2 generation DEGs are listed in **Table S2**). The unsupervised hierarchical clustering analysis of these DEGs, as visualized in heat maps, is shown in **Figure 4**. Overall assessment of the heat maps revealed that the expression pattern of the DEGs was mainly downregulated in the air-control groups, especially in the F2 generation, while both the F1 and F2 hypoxia groups primarily depicted upregulated expression. It is important to mention here that the differences between the three replicate samples of each group were small, thereby indicating good repeatability and a high confidence in the identified DEGs.

### Comparative Analysis of DEGs Identified in F1 and F2 Oocytes

**Figure 5:**
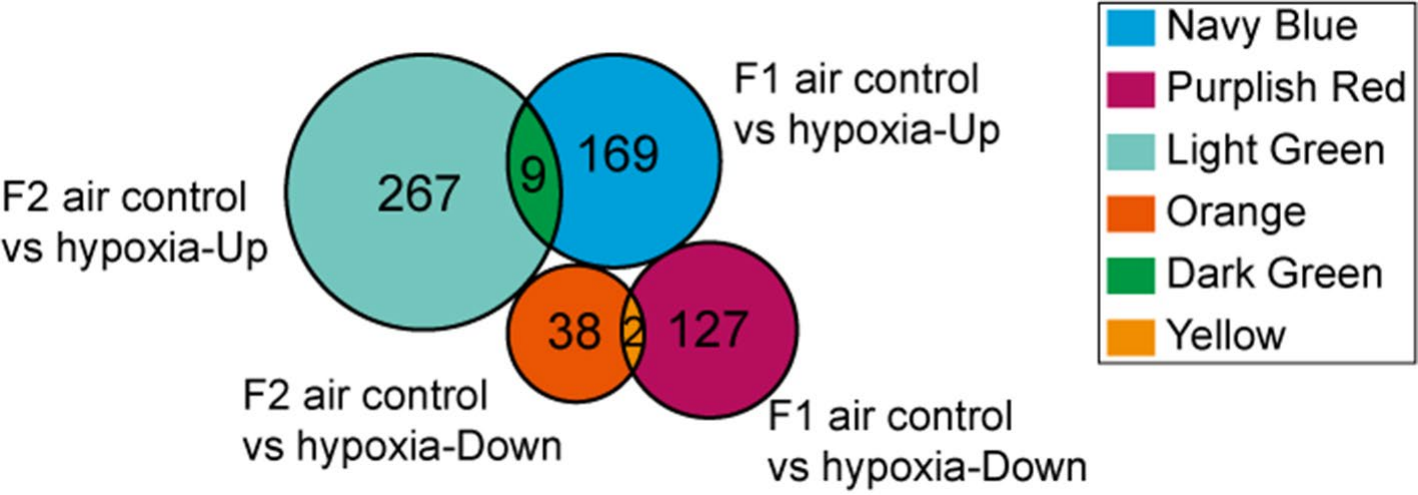
Venn diagram showing “Heredity” DEGs: Among the 296 genes classified as “Loss,” 169 were up-regulated (navy blue part) and 127 were down-regulated (purplish red part). Similarly, among the 305 genes defined as “Gain” 267 were up-regulated (light green part) and 38 were down-regulated (orange part). Overall, only 11 DEGs (“Heredity”) were identified to be expressed in both the F1 and F2 generations and had a similar trend of gene expression (dark green and yellow part).

DEGs found only in the F1 groups were defined as “Loss,” while those identified only in the F2 groups were defined as “Gain.” Moreover, genes that were differentially expressed in both generations (F1 and F2), with a similar trend of gene expression, were defined as “Heredity.” The overall aim of this study was to identify “Heredity” genes (**Table 2**). Venn diagram analysis was used to observe the inheritance of DEGs in the F1 and F2 groups which means the “Heredity” (**Figure 5**). From **Figure 5**, only nine up-regulated and two down-regulated DEGs were both found in the F1 and F2 generations and showed the same trend, which were defined as the “Heredity.” So there were 169 up-regulated and 127 down-regulated DEGs that were not changed in the F2 generation, which were defined as “Loss.” In addition, there were 267 up-regulated and 38 down-regulated DEGs that were newly emerged in F2 generation, which were defined as “Gain.” These “Heredity” genes were mainly involved in the composition of important organelles, like the nucleus, Golgi, endoplasmic reticulum, and lysosomes. GO term analysis indicated that these genes may also be involved in metabolic processes, such as lipid and protein metabolism, and may also participate in type II diabetes, insulin resistance, RNA polymerase, purine, arginine, proline, and glutathione metabolic pathways.

**Table 2:** Heredity DEGs.

Gene name	Up/down	Description	GO term	KEGG pathway	F1		F2	
					Fold-change	FDR	Fold-change	FDR
Cdk2ap1	Up	Cyclin-dependent kinase 2-associated protein 1	Nucleus	/	0.04	1	43.6	1
Ccdc91	Up	Coiled-coil domain containing 91	Membrane/Golgi apparatus	/	3.14	1	3.69	1
Odc1	Up	Ornithine decarboxylase 1	Cytoplasm/regulation of protein catabolic	Arginine and proline metabolism/glutathione metabolism	7.12	1	59.82	1
Dcaf15	Up	DDB1 and CUL4-associated factor 15	/	/	Inf	0.93	21.80	1
Laptm4b	Up	Lysosomal protein transmembrane 4 beta	Endomembrane system/protein binding	Lysosome	7.82	1	48.62	1
Polr2d	Up	Polymerase (RNA) II (DNA directed) polypeptide D	Transcription initiation from RNA polymerase II promoter	Purine metabolism/RNA polymerase	Inf	1	Inf	0.81
Prkcd	Up	Protein kinase C, delta	Protein kinase activity	Type II diabetes mellitus/insulin resistance	5.45	1	12.96	1
Gorasp1	Up	Golgi reassembly stacking protein 1	Golgi apparatus	/	Inf	1	Inf	1
Cisd2	Up	CDGSH iron sulfur domain 2	Mitophagy	/	2.51	1	2.94	1
Sult1d1	Down	Sulfotransferase family 1D, member 1	Metabolic process/lipid metabolic process	/	0.03	0.76	0	0.73
AABR 07013288.4	Down	/	/	/	92.24	0.76	0.38	1

DEG, differentially expressed gene; FDR, false discovery rate; GO, gene ontology; Inf, short for infinity; KEGG, Kyoto Encyclopedia of Genes and Genomes.

### GO Analysis of DEGs

**Figure 6:**
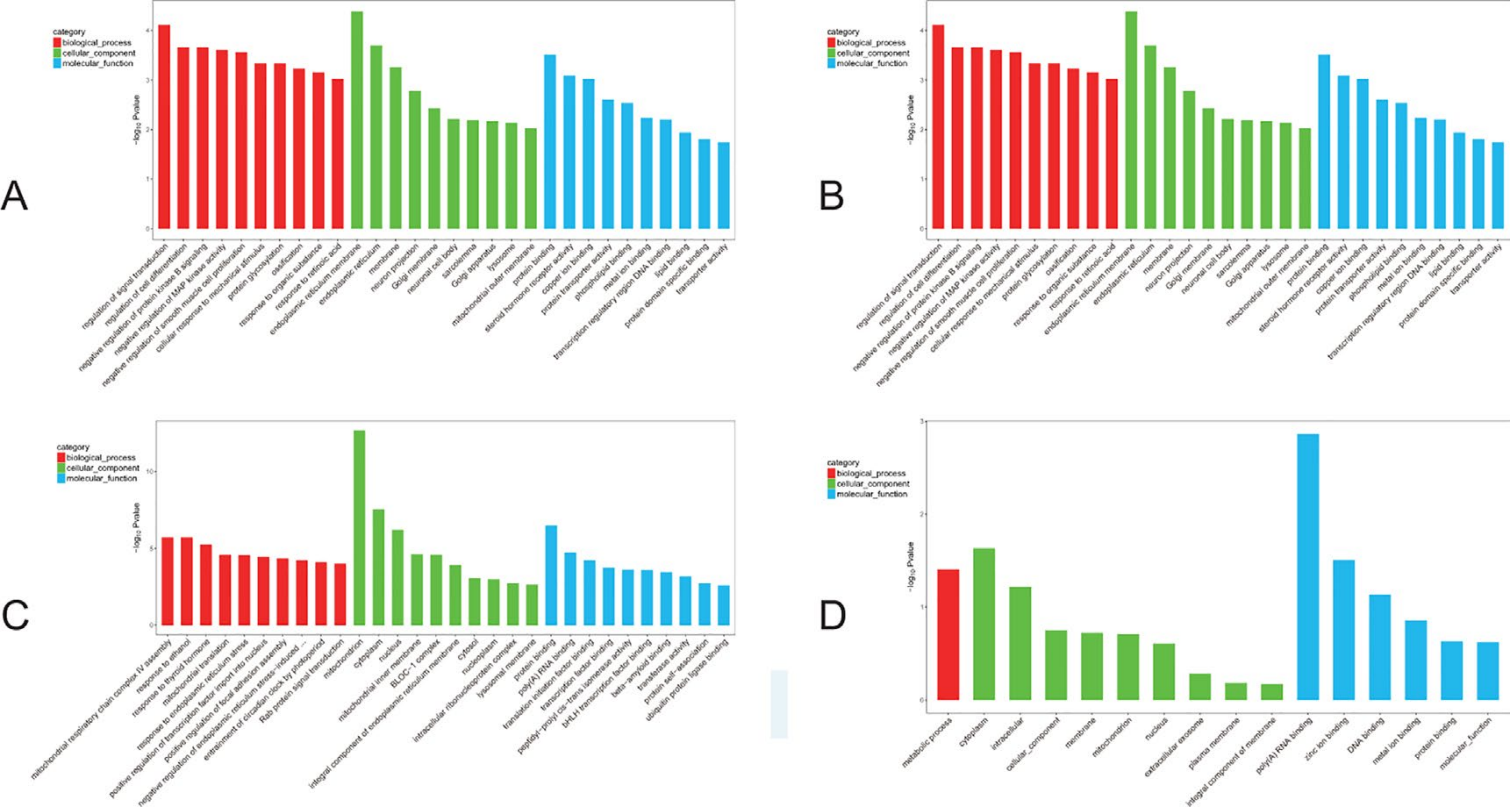
GO analyses of DEGs. The red bar: biological process; the green bar: cellular component; the blue bar: molecular function. **(A)** The GO analysis of the top 10 F1 up-regulated DEGs. **(B)** The GO analysis of the top 10 F1 down-regulated DEGs. **(C)** The GO analysis of the top 10 F2 up-regulated DEGs. **(D)** The GO analysis of the top 10 F2 down-regulated DEGs.

Further GO analysis of the DEGs was performed with terms involved in the biological process, cellular component, and molecular function. The top 10 genes of every significant GO category with a P-value < 0.05 are listed. The -log10) P value was used to describe the significance level of GO enrichment. A lower P-value correlated with a more significant GO term (**Figure 6**). Specifically, in the F1 generation, we found that upregulated DEGs were mainly involved in regulation of signal transduction as well as endoplasmic reticulum membrane and protein binding (**Figure 6A**); while cellular responses to mechanical stimuli, postsynaptic membranes, and drug binding were downregulated (**Figure 6B**). In parallel, for the F2 generation, we found that upregulated DEGs were mainly involved in the regulation of mitochondrial respiratory chain complex IV assembly as well as mitochondrion and protein binding (**Figure 6C**); while downregulated DEGs showed involvement in metabolic processes, the cytoplasm, and poly(A) RNA binding (**Figure 6D**).

### KEGG Pathway Analysis of DEGs

**Figure 7:**
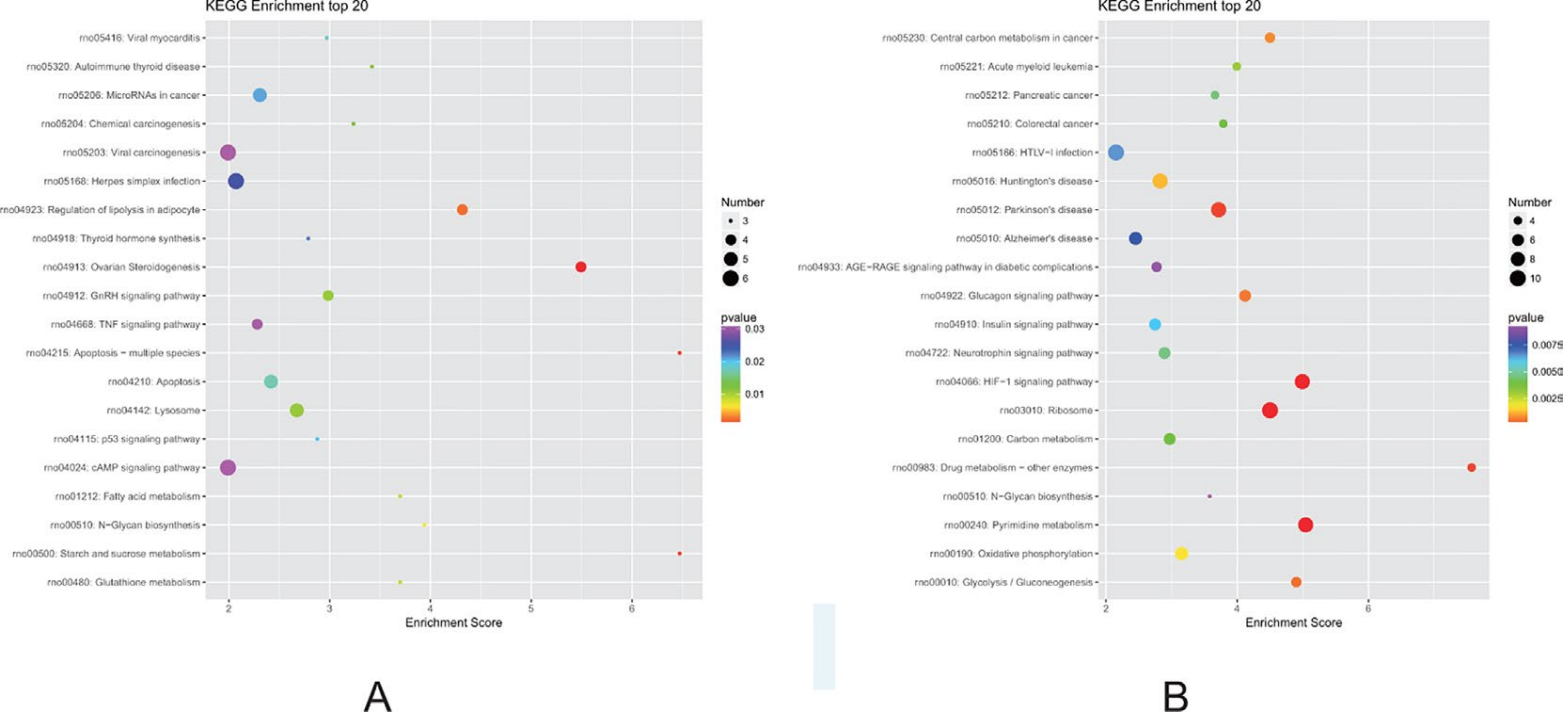
Kyoto Encyclopedia of Genes and Genomes (KEGG) pathway analysis for DEGs. **(A)** Pathways corresponding to DEGs from the air-control F1 versus hypoxia F1 rats. **(B)** Pathways corresponding to DEGs from the air-control F2 versus hypoxia F2 rats.

Finally, we performed KEGG pathway analysis of the identified DEGs. A higher enrichment score correlated with significant pathway regulation (**Figure 7**). In the F1 generation, the most enriched pathway observed was “starch and sucrose metabolism,” comprising three DEGs. In addition, it was also noted that the “apoptosis,” “regulation of lipolysis in adipocytes,” and “fatty acid metabolism” pathways may also play a key role in perinatal hypoxia stress (**Figure 7A**). However, in the F2 generation, the most enriched pathway was “drug metabolism,” comprising four DEGs. Furthermore, the “HIF-1 signaling pathway,” “oxidative phosphorylation,” and “glycolysis” pathways also were associated with DEGs between the F2 groups (**Figure 7B**).

## Discussion

In this study, we performed single-cell sequencing analysis of oocytes isolated from rats treated with intrauterine hypoxia and their offspring. Our results demonstrated that intrauterine hypoxia can potentially cause alterations in the gene expression of female gametes in future generations, as analyzed by the transcriptome sequencing technique. In addition, some gene expression changes were also observed to be transmitted to the F2 generation oocytes, despite the F2 generation not experiencing any intrauterine hypoxia. Thus, these findings indicate that the foundation of some adult diseases, such as hypertension and diabetes mellitus, etc., could be due to perinatal environment-induced gene expression. Currently, we do not know the exact mechanism of induction of these changes in gene expression. However, there are two novel aspects of our findings; 1) while most previous studies have focused on epigenetic marker changes in different organs, our study directly identified an alteration in the gene expression of oocytes, 2) our transcriptome sequencing data revealed 11 DEGs that were inherited to the next-generation oocytes, even though the next generation was not directly exposed to the hypoxic environment.

Intrauterine hypoxia, the most common complication during pregnancy, can be caused by smoking (Hickey et al., 1978), maternal anemia (Soares et al., 2017), or eclampsia (Ramjiawan and Tappia, 2018). To analyze the effect of antenatal hypoxia specifically, we used the previously established intrauterine-hypoxia model (Li et al., 2017). In recent years, there have been growing concerns about the effects of the environment early in life toward later in life development (Ramjiawan and Tappia, 2018; Schepanski et al., 2018). Intrauterine hypoxia has been shown to cause intrauterine growth retardation (Malhotra et al., 2019), decreased brain adaptive potential (Nalivaeva et al., 2018), and even an increased rate of stillbirth. However, the impact of antenatal hypoxia on the offspring is not restricted to the perinatal period or only the following generation. A similar phenomenon was also observed for the onset of diabetes mellitus (Aerts et al., 2010).

The question of why the phenomenon occurs still cannot be explained clearly. The classic inheritance of phenotypes from our forebears to the offspring is usually mediated by copying and transmission of the DNA sequence. However, due to the redundancy and stability of the genome as well as genomic protection mechanisms like gene ubiquitination modification and DNA repair, most environmental factors cannot directly lead to DNA mutations. Thus, the mechanism of transgenerational or multigenerational inheritance may involve epigenetic alteration (Nilsson et al., 2018). Other studies have focused on epigenetic changes in specific organs after intrauterine adverse environmental exposure. For instance, it was observed that a low-protein diet during pregnancy could lead to alteration of the levels of insulin-like growth factor, leptin, and peroxisome proliferator-activated receptor gamma in the liver tissues of the offspring (Lillycrop et al., 2008). But how the epigenetic alterations of genes in organs can be passed to the next generation is still unknown. Previous study (Mohan et al., 2013) found that kidney and prostate disease were only observed in the directly exposed F1 generation animals but the methylation analysis of the sperm provided evidence for transgenerational inheritance pattern casued by plastic compounds. So our study focused on the female gametes.

Back in 1997, one of the first studies about epigenetic inheritance in mice showed that some specific changes, like reduced weight or alteration of gene expression, were transmitted to the majority of the offspring of manipulated parent mice (Roemer et al., 1997). This was the first demonstration of epigenetic inheritance, pointing to the focus on the germline. Another study using sperm DNA sequencing revealed that hypomethylation of the Olfr151 gene (an acetophenone receptor gene) may be an essential part of transgenerational inheritance of the sensitivity to acetophenone in mice (Virginia, 2014). However, in clinical situations, it is usually the mother who experiences most adverse stress during pregnancy. So, our assumption was that single-cell sequencing of oocytes may provide a novel way to understand the basis of multigenerational or transgenerational inheritance. However, among the five epigenetic processes (Nilsson et al., 2018) that can impact gene expression, it was difficult and cumbersome to analyze the effects of individual processes. Therefore, we decided to perform transcriptome sequencing on oocytes first to analyze the alteration in transcriptional level, irrespective of the five epigenetic processes.

Another theory of “fetal origins in adult disease” was proposed by Dr. Barker (Barker et al., 2002), and his epidemiological investigations revealed that type 2 diabetes mellitus (Barker et al., 1993), hypertension (Barker et al., 1993), and hyperlipidemia (Barker et al., 1993) were all related to reduced fetal growth. There is also emerging evidence pointing to the fact that initiation of cardiovascular and metabolic disorders in adult life may be related to adverse insults during fetal development (Longo, 2013). Consistent with this idea, our GO-term and KEGG-pathway results also demonstrated that 11 DEGs mainly participated in the type II diabetes mellitus and insulin resistance pathway, along with their involvement in metabolic or lipid metabolic processes. Our team had already found that the antenatal hypoxia could cause the increased susceptibility to diabetes induced by a high-fat diet in the offspring rats (Huang et al., 2017). These results further confirm the genetic basis of some diseases. In addition, we also noted that “heredity” DEGs are involved in the composition of important organelles such as the nucleus, Golgi apparatus, endoplasmic reticulum, and lysosome. The nucleus, which contains most of the genetic material, is a membrane-enclosed organelle found in most eukaryotic cells. Similarly, the Golgi apparatus, endoplasmic reticulum, and lysosome are all part of the endomembrane system. Thus, the possible mechanism of multigenerational inheritance might involve these organelles. Moreover, the molecular function of the GO term pointed toward the “protein binding” and “regulation of protein catabolic” terms. This finding implied that protein-related function may also play an important role in epigenetic inheritance. It is also worth noting that the gene Cisd2 is related to mitophagy, which is the selective degradation of mitochondria by autophagy and is an essential part of mitochondrial quality control. An imbalance of mitophagy can lead to cell death, eventually resulting in organ dysfunction. Previous studies regarding Cisd2 mostly focused on type 2 Wolfram syndrome, a rare neurodegenerative and metabolic disorder associated with a shortened lifespan (Urano, 2016). Thus, it is highly possible that the onset of some adult diseases like metabolic syndrome may be caused by an alteration in Cisd2 expression. It is also important to mention here that most previous studies on the identified 11 DEGs showed their association with the cell cycle or cell growth and proliferation. Cdk2ap1, one of the inherited genes, is regarded as one of the cell-cycle regulator, which is identified as a growth suppressor. Most studies of Cdk2ap1 are involved with cancer (Olga and Figueiredo, 2010; Li-Ching et al., 2012). Previous study (Wong et al., 2012) has confirmed that Cdk2ap1 is expressed in the early stage of the pre-implantation embryos and may be associated with the epigenetic control.On the contrary to the Cdk2ap1, the Laptm4b can promote the growth and proliferation of cells in various kinds of tumors (Gen-Ze et al., 2003; Mingzhu et al., 2011). Ccdc91 are quite rare. Sprooten et al. used the co-analysis of the DTI data and RNA transcripts derived from lymphocytes and the results showed that the expression of the Ccdc91 was associated with the brain image, suggesting that the protein product of the Ccdc91 may be involved in white matter microstructure (Sprooten et al., 2014), which indicates the connection of the Ccdc91 and the brain development. Ornithine decarboxylase 1 (ODC1) is the rate-limiting enzyme in endogenous polyamine synthesis. Polyamine metabolism is involved in cell proliferation, differentiation, and other important biological processes through the regulation of gene expression. Choi Y’s research mentioned the up-regulation of the ODC1 in hepatocellular carcinoma and inhibiting the ODC1 can decrease the growth and increase the apoptosis (Choi et al., 2016). Another interesting finding is that the odc1 silencing can inhibit the glucose transport and lipid biogenesis (Choi et al., 2016). Although we don’t the effect of the up-regulation of the ODC1 toward glucose and lipid metabolism, we at least can conclude that the ODC1 might associate with the glucose and lipid metabolism.Studies about the DCAF15 are focused on cancer and the target of the anti-cancer drug like the sulphonamides (Han et al., 2017). The gene Prkcd is thought to be involved with the development of the type 2 diabetes and obesity. Olivier Bezy’s research found that Prkcd could regulate the development of the insulin resistance and increased expression of the gene was an important feature of the pro-diabetes mice (Vernochet et al., 2012). Gorasp1 is also known as GRASP65, one of the Golgi re-assembly and stacking protein in mammalian cells. According to previous study (Danming and Yanzhuang, 2013), depletion of the Gorasp1 could reduce the number of cisternae per stack, indicating that the gene might play an important role in the structure of the Golgi. During cell cycle, the phosphorylation and dephosphorylation of the gene can directly influence the protein trafficking and Golgi reassembly.

Importantly, our study demonstrated that only a small portion of DEGs were inherited from the F1 to F2 generation. Several factors may contribute to this phenomenon, including the following: 1) the F2 generation not only inherits genetic material from their mother (F1 generation), but it also inherits some genetic material carried from the sperm of their father. In other words, the genetic information of the oocytes (F2 generation) is regulated by both parents; 2) During the early fertilization and embryonic formation processes, the epigenetic markers from the parents undergo reprogramming; therefore, the methylation state of the epigenetic markers is erased and re-established. Only a small number of genes can avoid this process and eventually influence the phenotype of the offspring; 3) The gene expression regulation is also affected by many other factors, including a change in the environment. Since the oocytes of the F2 generation were not exposed to the hypoxic environment just like their mother (F1 generation), they would adapt differently to the normal environment, ultimately resulting in a gene expression pattern that is not the same as that of their mother (F1 generation).

Finally, some limitations of this study must be mentioned. First, we did not explore the exact mechanism of altered gene expression. Since methylation is one of the most widely investigated epigenetic process., in future studies, we will perform methylation sequencing and transcriptome sequencing of oocytes in parallel. Second, due to the difficulty in obtaining oocyte samples, it was difficult to validate our RNA-seq results. The majority of the studies about the single-cell sequencing involving the oocyte, embryo, blasomere, and embryonic stem cell, these reports didn’t perform the validation by RT-PCR (Zhigang et al., 2013; Yan et al., 2013). Additional, validation of findings at the protein level might be another approach. Combining different methods to validate the sequencing results is our future plan. Thirdly, we didn’t consider the sensitivity of the superovulation effect on the control and hypoxia group. Whether the superovulation procedure could change the oocytes’ gene expression is still controversial. One of the previous studies showed that the superovulation couldn’t disrupt the maternal imprint acquisition in oocytes when compared with the spontaneously ovulated females (Denomme et al., 2011). On the contrary, Stouder C fould that superovulation in the mother transgenerationally affects the offspring sperm methylation patten (Stouder et al., 2009). In addition, although the abnormal gene expression in oocytes induced by intrauterine hypoxia has not been confirmed by other previously published studies, it has been suggested that exposure to intrauterine hypoxia can lead to abnormal gene expression in rat oocytes and that some abnormal genes can be inherited to the next generation. Thus, additional studies are required to explore the relationship between gene expression changes and their long-term effects on organ function.

## Conclusion

In summary, our study is the first report showing gene expression alteration in oocytes of rats under intrauterine hypoxia and their offspring, as analyzed by single-cell transcriptome sequencing. We identified 11 DEGs that were inherited from the F1 to F2 generation. These DEGs were specifically enriched in metabolic processes like lipid and insulin metabolism. Taken together, it is evident that the alteration in gene expression of oocytes may be associated with some metabolic diseases in the future and that it could be the basis of transgenerational or multigenerational inheritance induced by an adverse perinatal environment.

## Data Availability Statement

The datasets analyzed for this study can be found in the SRA NCBI. The accession numbers are SRR9425429 (F1generation-sampleB1), SRR9425428 (F1generation-sampleB3), SRR9425427 (F1generation-sampleA1), SRR9425426 (F1generation-sampleA2), SRR9425425 (F1generation-sampleA3), SRR9425424 (F1generation-sampleB2), SRR9221191 (F2genetation-sampleA1), SRR9221192(F2genetation-sampleB3), SRR9221193 (F2genetation-sampleB1), SRR9221194 (F2genetation-sampleB2),SRR9221195 (F2genetation-sampleA3), and SRR9221196 (F2genetation-sampleA2).

## Ethics Statement

The animal study was reviewed and approved by Committee of Research Animal Welfare, Central South University, Changsha, China.

## Author Contributions

SY and ZCL conceived and designed the whole experiment together. TL established the animal model and finished the following sequencing. YL contributed to the analysis and interpretation of results and development of the manuscript. ZQL helped with some technical problems during the whole experiment. MW, CC, YD, and ZLL assisted the statistical analysis and graphical representation. All authors reviewed and improved the final version of the manuscript.

## Funding

Funding was provided by National Science Foundation of China (Grant No. 81370098 and No. 81801510).

## Conflict of Interest

The authors declare that the research was conducted in the absence of any commercial or financial relationships that could be construed as a potential conflict of interest.

## Supplementary Material

The Supplementary Material for this article can be found online at: https://www.frontiersin.org/articles/10.3389/fgene.2019.01102/full#supplementary-material

Table S1 DEGs of F1 generation (Air control versus hypoxia group).

Table S2 DEGs of F2 generation (Air control versus hypoxia group).
